# A case of hypoparathyroidism with cardiovascular complications: interventricular septal dissecting aneurysm in a middle-aged woman

**DOI:** 10.1186/s12902-025-02009-9

**Published:** 2025-07-25

**Authors:** Ronghua Huang, Yi Zhou, Xingshou Pan, Liufang Zhou, Zhengjiang Liu

**Affiliations:** Department of Cardiology, Affiliated Hospital of Youjiang Medical University for Nationalities, Youjiang Medical University for Nationalities, Baise, 533000 Guangxi P.R. China

**Keywords:** Hypoparathyroidism, Interventricular septal aneurysm, Cardiovascular disease, Parathyroid hormone

## Abstract

**Background:**

Hypoparathyroidism (HypoPT) is a rare endocrine disease characterized by hypocalcemia, hyperphosphatemia, and insufficient or no parathyroid hormone (PTH) secretion. Hypoparathyroidism-induced chronic hypocalcemia may lead to cardiovascular complications, including myocardial dysfunction and arrhythmias. Interventricular septal dissecting aneurysm, a rare cardiac anomaly, typically arises following structural or ischemic heart disease. This disease can lead to ventricular septal rupture and aneurysmal cystic chamber formation, causing changes in cardiac structure and hemodynamics as well as giving rise to various heart complications.

**Case presentation:**

No study has reported an association between hypocalcemia and cardiac masses. We encountered a case of hypoparathyroidism in a middle-aged woman who developed an interventricular septal dissecting aneurysm. Upon examination, we found that HypoPT has a profound impact on the heart.

**Conclusions:**

Hypocalcemia caused by HypoPT can result in structural changes in the heart and myocardial injury. Furthermore, there is a potential link between HypoPT and cardiac aneurysm. The development of an interventricular septal dissecting aneurysm can lead to various cardiac complications; therefore, its early diagnosis and treatment are necessary.

## Background

Hypoparathyroidism (HypoPT) is a rare syndrome caused by insufficient parathyroid hormone (PTH) secretion. It is characterized by low serum calcium levels associated with insufficient PTH levels, changes in the phosphate/PTH balance that can lead to vascular calcification, damage to accompanying or nonaccompanying reactions, and increased neuromuscular excitability and soft tissue calcification. These changes increase the risk of cardiovascular disease and mortality [1–3]. Calcium is indispensable for the proper functioning of the heart; it is not only involved in the contraction and relaxation of the fibers but also in the generation of the action potential, indicating the relationship between hypocalcemia and electrocardiographic abnormalities (QT interval prolongation) and ventricular dysfunction. HypoPT-induced hypocalcemia can lead to complications such as myocardial fibrosis and degeneration. Chronic hypocalcemia may lead to electrocardiographic (ECG) changes and mimic acute coronary syndrome [4].

Interventricular septal dissecting aneurysm is an extremely rare cardiovascular disease that typically arises as a complication of other diseases. It occurs when the interventricular septum is damaged, leading to interventricular septal rupture and aneurysmal cystic cavity formation. Moreover, this disease can lead to changes in the structure and hemodynamics of the heart. Interventricular septal dissecting aneurysms have been reported to occur following aortic sinus aneurysm rupture, infectious endocarditis, cardiac surgery, trauma, acute myocardial infarction, congenital coronary artery fistula, pericardiocentesis, and balloon angioplasty; furthermore, it is frequently associated with aortic sinus aneurysm [5–7]. In this study, we report a case of HypoPT-induced hypocalcemia accompanied by a ventricular septal dissection aneurysm in a middle-aged woman.

## Case presentation

**Fig. 1:**
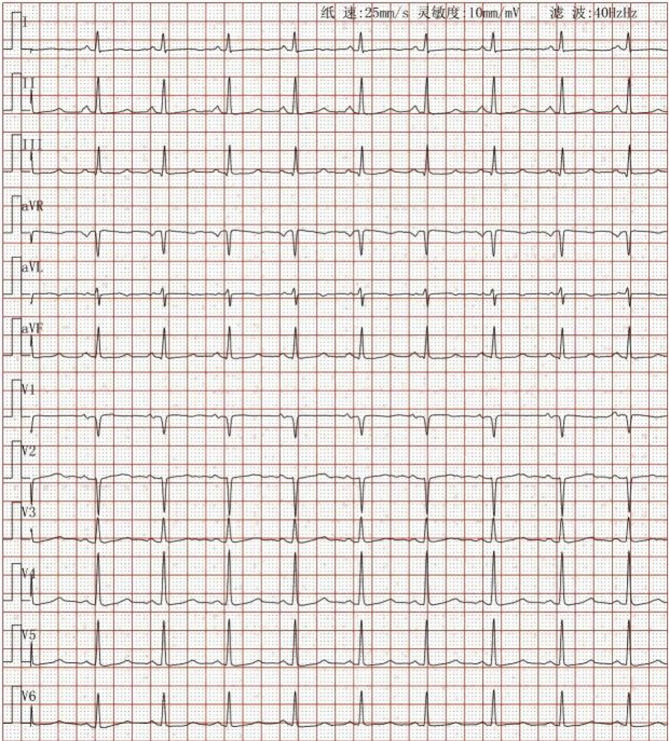
An electrocardiogram showing sinus rhythm, ST–T changes and a long QTc interval

**Fig. 2:**
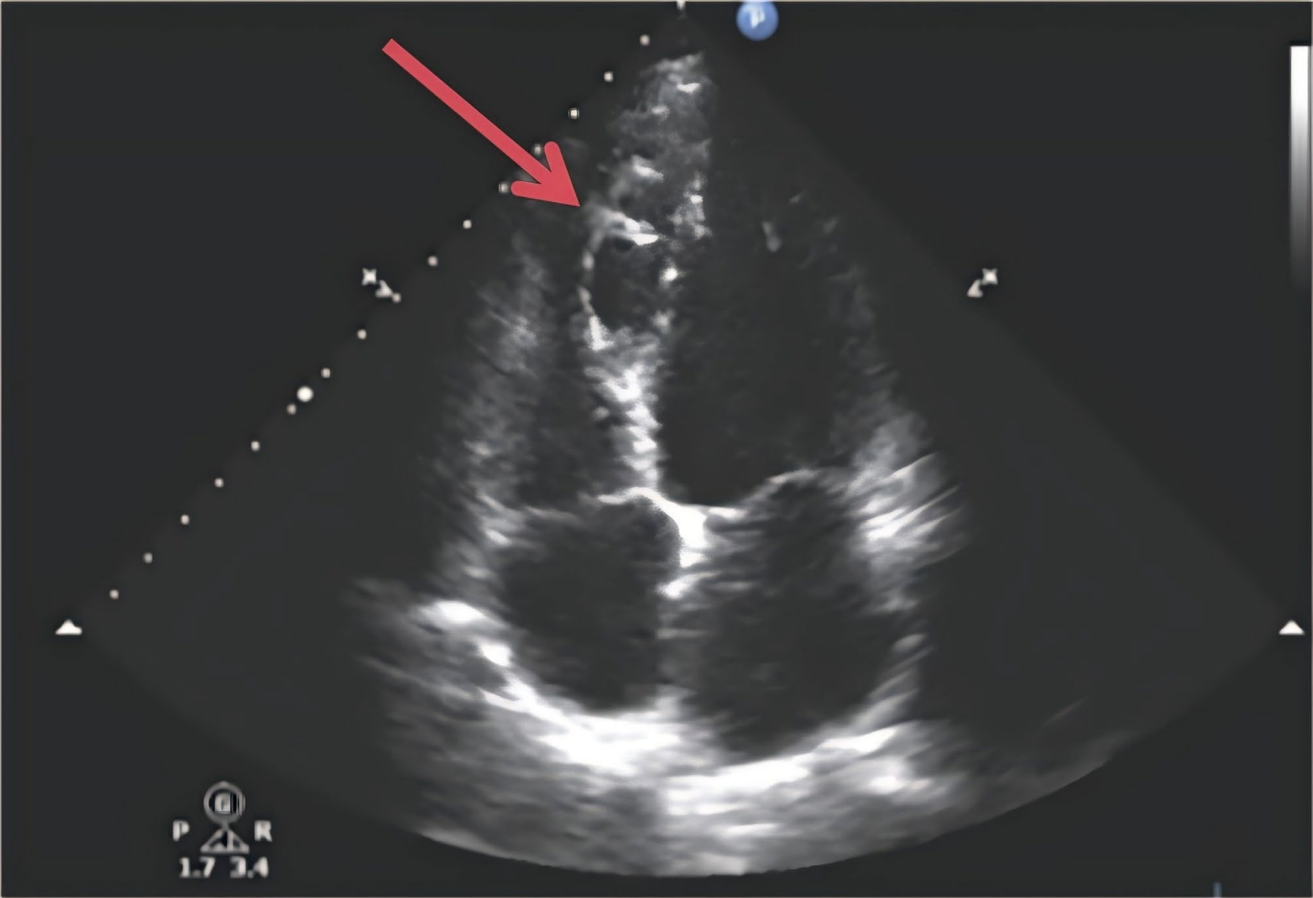
A cardiac echocardiogram showing ventricular septum-inferior wall dissecting aneurysm formation. An irregular anechoic chamber (18 mm wide) communicating with the ventricular cavity was detected between the lower segment of the interventricular septum and the inferior wall

**Fig. 3:**
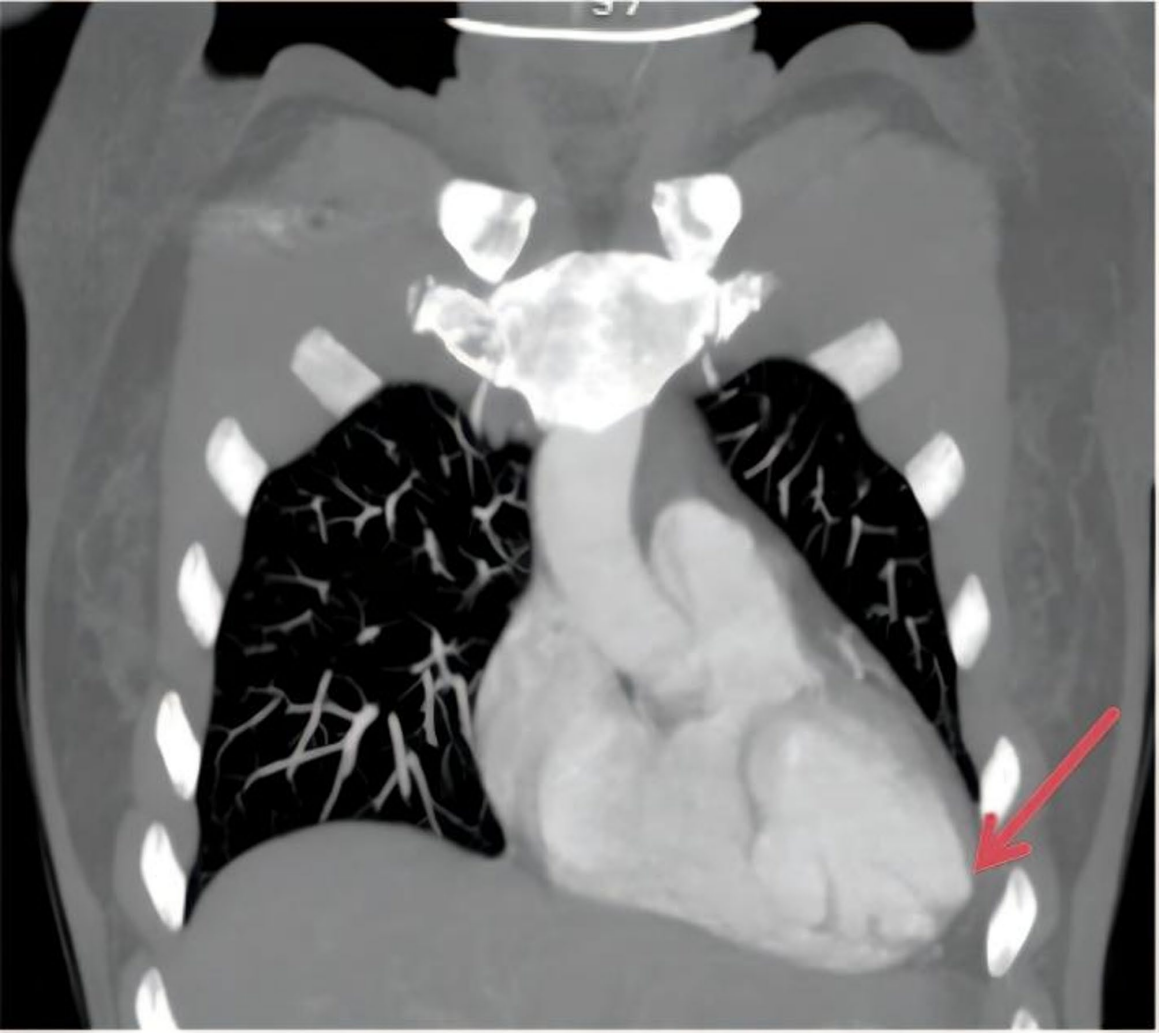
CTA of the thoracic aorta revealed ventricular septum-inferior wall aortic dissecting aneurysm formation.Multiple cystic protrusions were present in the interventricular septum and inferior wall, and no contrast agent overflow was observed in the pericardial cavity

**Fig. 4:**
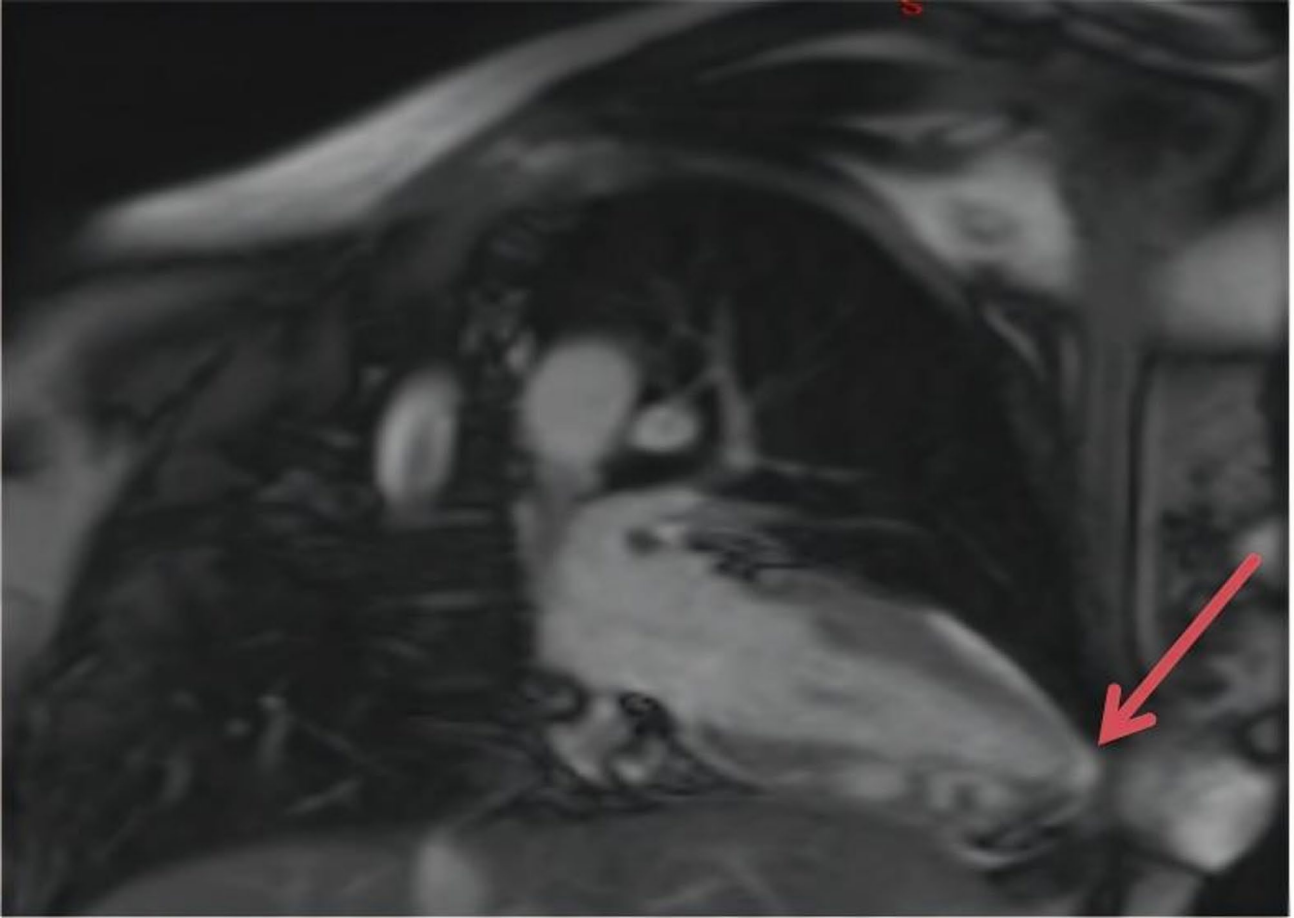
Cardiac MRI showing a ventricular septal dissecting aneurysm. An abnormal signal mass of approximately 16 mm × 42 mm in size partially communicated with the left ventricular cavity in the inferior wall of the interventricular septum

A 45-year-old woman was admitted to our hospital because of repeated paroxysmal chest tightness and palpitations for over a year, which worsened during the last 5 h. The patient reported experiencing chest tightness and palpitations over a year ago without obvious triggers, primarily presenting as paroxysmal precordial distension that lasted approximately 3–5 min per episode, with no clear alleviating or aggravating factors. In the past 5 h, the chest tightness symptoms significantly worsened, accompanied by palpitation discomfort, numbness in the limbs, and tingling sensations. She had a history of thyroid surgery, hyperlipidemia, and hospitalization for numbness and weakness of the limbs more than 10 years before presentation; however, the specific details were not available. She denied living in an epidemic area or having contact with dogs in the past. Physical examination revealed that her pulse was 81 beats per minute, and she had clear consciousness, with a surgical scar of approximately 5 cm in length on the neck. Furthermore, she had dry and flaky skin on the hands and feet, clear breath sounds in both lungs, no dry or moist rales, and no enlarged heart borders. Her heart rate was 81 beats per minute, and she had a regular heart rhythm, with no murmurs heard in any valve auscultation area. She tested positive for the Chvostek and Trousseau signs. Moreover, no significant abnormal findings were observed in pulmonary, abdominal, and neurological examinations. After admission, the following parameters were measured and recorded: low-density lipoprotein cholesterol, 4.63 mmol/L; apolipoprotein B, 1.20 g/L; calcium, 1.42 mmol/L; phosphorus, 1.74 mmol/L; vitamin D3, 17.42 ng/mL; PTH, < 1.000 pg/mL; bone gamma-carboxyglutamic acid, 20.29 ng/mL; serum calcitonin, 7.11 pg/mL; lactate dehydrogenase, 362 U/L; creatine kinase, 320 U/L; and creatine kinase isoenzyme, 17.8 U/L (Table  1). Routine blood, thyroid function, autoimmune rheumatism, hydatid cyst fluid antigen, and indirect hemagglutination tests; myoglobin, troponin, and plasma d-dimer assays; and other biochemical tests revealed no abnormalities. Meanwhile, electrocardiography revealed sinus rhythm, ST–T changes and a long QTc interval (Fig. 1). Thyroid ultrasound revealed a left thyroid mixed nodule (C-TIRADS grade 3). No significant abnormality was detected in the bilateral parathyroid areas, but primary HypoPT could not be excluded. Cardiac ultrasound revealed an internal echolucent chamber in the ventricular septum-inferior wall, suggesting the possibility of dissecting aneurysm formation without excluding congenital possibilities. Thus, further examination was recommended. Left ventricular systolic function was normal (Fig. 2). Computed tomography angiography (CTA) of the aorta and abdominal aortic revealed that the ventricular septum-inferior wall aneurysm was possibly a dissecting aneurysm (Fig. 3); moreover, stenosis of the abdominal aortic was observed. No abnormalities were detected on whole-abdomen computed tomography. Parathyroid and myocardial imaging indicated no findings of hyperparathyroidism and reduced blood flow in the left ventricular anterior interventricular septum myocardium. Cardiac magnetic resonance imaging (MRI) identified possible ventricular septum-inferior wall dissecting aneurysm formation (Fig. 4). Coronary angiography revealed diffuse sclerosis and stenosis of approximately 30–40% of the proximal-to-middle segment of the left anterior descending artery and limited stenosis of approximately 50% of the middle segment of the left circumflex artery and right coronary artery. Blood flow was TIMI grade 3 in all areas, with no other significant stenosis identified.

**Table 1:**
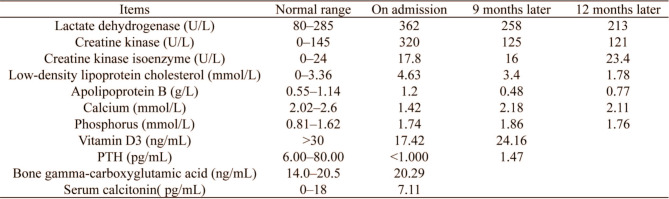
Laboratory findings of a middle-aged woman with hypoparathyroidism

After evaluating the surgical risks, we found it suitable to perform volume reduction surgery for ventricular septal dissecting aneurysm under general anesthesia, hypothermia, and extracorporeal circulation. However, the patient refused to undergo heart surgery and was administered symptomatic and supportive treatments, such as antiplatelet aggregation (Clopidogrel bisulfate tablets: 75 mg/day, oral administration), plaque stabilization (Atorvastatin: 20 mg/day, oral administration), and calcium supplementation (Calcitriol Soft Capsules: 0.5 µg/day, Calcium Carbonate D3 Tablets: 600 mg/day, oral administration). Following these treatments, the patient’s condition improved, and she was discharged. At an outpatient follow-up 9 months after discharge, her serum calcium level was 2.18 mmol/L, vitamin D3 level was 24.16 ng/mL, and PTH level was 1.47 pg/mL (Table  1). No changes were observed in cardiac ultrasound and thoracoabdominal aortic CTA findings, and no episodes of chest tightness or palpitations occurred. The patient continued the medicines (Calcitriol Soft Capsules: 0.5 µg/day, Calcium Carbonate D3 Tablets: 600 mg/day, oral administration), and at an outpatient follow-up 1 year later, her serum calcium level was 2.11 mmol/L (Table  1). She continued the medicines (Calcitriol Soft Capsules: 0.5 µg/day, Calcium Carbonate D3 Tablets: 600 mg/day, oral administration) and remained in stable condition.

## Discussion and conclusions

HypoPT is a rare form of parathyroid disease characterized by insufficient PTH levels, resulting in low serum calcium and high serum phosphorus levels [8]. The incidence of HypoPT is approximately 1–3 per 10,000 people, with most cases associated with previous neck surgery, including total thyroidectomy [9]. Patients with postoperative or nonsurgical chronic HypoPT who are treated with conventional therapy are at an increased risk of developing various cardiovascular diseases and complications, including arrhythmia; increased carotid artery intimamedia thickness, stiffness, and calcification; carotid artery calcification; coronary artery disease; cardiovascular autonomic neuropathy; and heart failure. Elevated or low levels of calcium and PTH are associated with increased mortality from cardiovascular disease [10, 11]. Interventricular septal dissecting aneurysm is a rare clinical condition that can lead to various cardiac complications such as aortic valve regurgitation, left and right ventricular outflow tract obstruction, and various arrhythmias [12, 13]. It can also occur as a rare complication of acute myocardial infarction, with myocardial dissection being a special type of subacute cardiac rupture manifestation [14–17]. It is characterized by the rupture of endocardial muscle fibers, entry of blood into the spiral interphase of the myocardium, and formation of dissection and hematoma; however, the epicardial muscular fibers remain intact [18]. If an interventricular septal dissecting aneurysm forms a dissection without rupture and is small in size, it might form a thrombus and shrink, thus requiring follow-up observation. Rupture of interventricular septal dissecting aneurysms is extremely dangerous and fatal. Notably, cardiac masses associated with HypoPT have not been reported in the literature. To the best of our knowledge, the present case is the first report of the occurrence of HypoPT following cervical thyroidectomy due to possible resection, injury, or vascular dysfunction of the parathyroid glands, resulting in insufficient PTH production. In the present case, the long-term effects of inadequate parathyroid reserve were low calcium and PTH levels and loss of phosphorylating activity, which resulted in high phosphorus content and vitamin D deficiency. The clinical manifestations were limb numbness and chest discomfort. Auxiliary examination revealed a series of cardiovascular disease manifestations, such as a prolonged QT interval on electrocardiogram and stenosis of the initial part of the celiac artery. The cardiac blood supply was unique, with the majority of the myocardium being perfused during diastole. We observed that coronary artery spasm due to hypocalcemia could lead to ischemia if the diastolic myocardial blood flow decreased below a critical threshold, thereby causing a supply–demand mismatch. This could be worsened by HypoPT-induced hypocalcemia and lead to complications such as myocardial fibrosis and degeneration. Based on these findings, we suspected myocardial infarction, a condition similar to ST-segment depressed myocardial infarction on electrocardiogram [19, 20]. Myocardial rupture led to the penetration of the ventricular septum, resulting in shunting and ventricular dissection. Myocardial fibers were separated by blood flow, forming tunnels or cysts. The left and right ventricles were not ruptured, which was limited to the interventricular septum and lower wall below the midpoint, forming an aneurysm [21]. In this case, cardiovascular magnetic resonance (CMR) imaging did not reveal any evidence of fibrosis. Similarly, in another study, CMR imaging did not reveal myocardial fibrosis in parathyroidectomized patients, which might be related to the duration and severity of hypocalcemia or to the patient’s compliance with prescribed medications and calcium supplements [22]. Further research is needed to understand the mechanisms involved.

Hypocalcemia caused by HypoPT can lead to arrhythmia and heart failure. The present case also indicated a potential link between hypoparathyroidism and cardiac aneurysm. Patients with HypoPT-induced hypocalcemia can develop a ventricular septal dissecting aneurysm, which can cause structural changes in the heart and myocardial injury, Further research is needed to clarify the mechanism. HypoPT has a profound impact on the heart, therefore, early diagnosis and treatment of HypoPT-induced hypocalcemia are necessary.

## Data Availability

No datasets were generated or analysed during the current study.

## Abbreviations

HypoPT: Hypoparathyroidism
PTH: Parathyroid hormone
QT: QT interval
ECG: Electrocardiographic
CTA: Computed tomography angiography
MRI: Magnetic resonance imaging
CMR: Cardiovascular magnetic resonance
